# Live-Cell Imaging of Microglia in Organotypic Brain Slices Using Microcontact Printing

**DOI:** 10.3390/biom16050713

**Published:** 2026-05-12

**Authors:** Björn Y. P. Richardsen, Christian Humpel

**Affiliations:** Laboratory of Psychiatry and Experimental Alzheimer’s Research, Department of Psychiatry and Psychotherapy, Medical University of Innsbruck, Anichstr. 35, A-6020 Innsbruck, Austria

**Keywords:** microglia, organotypic brain slice, live-cell imaging, microcontact print

## Abstract

Microglia are brain immune cells that phagocytose cell debris and beta-amyloid plaques in patients with Alzheimer’s disease. They develop from round amoeboid cells into ramified microglia or large macrophages, which can be studied in three-dimensional organotypic mouse brain slices. In a recent publication, we showed for the first time that we can track GFAP+ astrocytes and laminin+ vessels in organotypic brain slices using live-cell imaging . The aim of the present study was to use microcontact printing on organotypic brain slices to label microglia with Iba1 and CD11b antibodies and visualise them through live-cell imaging. We show that microglia can be easily labelled with antibodies and tracked via live-cell fluorescence microscopy for up to 20 days. Incubation in lipopolysaccharide (LPS) or granulocyte–macrophage colony-stimulating factor (GM-CSF) stimulates the migration of round amoeboid microglia, whereas interleukin-10 induces their differentiation into ramified forms. Taken together, we show the first-time live cell imaging of microglia in organotypic mouse brain slices using microcontact printing.

## 1. Introduction

Microglia are brain immune cells that originate from the mesoderm and play an important role in the central nervous system [1,2,3]. Their main immune function is to phagocytose toxic cellular debris and other pathogens [3,4]. Microglia release chemokines and cytokines like tumour necrosis factor alpha (TNF-α), interleukin-1β (IL-1β), and interleukin-10 (IL-10) and have either a pro-inflammatory role or anti-inflammatory activity [5,6,7]. There are three distinctive forms of microglia visible in the brain: those that are dependent on developmental, activation, or pathological processes, producing round amoeboid microglia, ramified-activated or non-activated resting microglia, and large macrophages, respectively [8].

Alzheimer’s disease (AD) is the most common form of dementia , and the main pathologies of sporadic AD are the deposition of extracellular β-amyloid plaques (Aβ) and intraneuronal Tau neurofibrillary tangles . It is well known that neuroinflammation is a pronounced pathology in AD . While microglia are activated in AD and phagocytose plaques, they are dysfunctional in severe AD stages [9,10,11,12,13,14,15,16].

Organotypic brain slices are three-dimensional, 150 μm thick sections derived from the brains of postnatal day 8–10 mice that can be cultured for several weeks in vitro (see detailed reviews [17,18,19,20]). Microglia survive in organotypic brain slices [21,22,23], and several additional papers have since shown this trait, allowing them to be studied [24,25]. In a previous study, we demonstrated that microglia migrate along microcontact-printed lanes loaded with monocyte chemoattractant protein-1 (MCP-1) [26], and we recently reported that microglia migrate along microcontact prints loaded with human plasma, which helped us to identify novel human AD biomarkers [27].

However, following living brain cells in organotypic brain slices ex vivo remains challenging. In a recent publication, we showed for the first time that we can track GFAP+ astrocytes and laminin+ vessels in organotypic brain slices using live-cell imaging [17]. To visualise these cells, we developed a novel and innovative technique in our lab: the microcontact printing (µCP) technique. This method has been described in detail [28], and we recently showed that, by using this technique, we are able to label brain cells directly in organotypic slices [17]. Briefly, we load an antibody into a collagen solution and print this antibody with a stamp (made from a master) directly onto the slice as 400 µm spots. The antibody is then labelled with a fluorescent secondary antibody, and the slices are incubated for several weeks, allowing the labelled cells to be stimulated and visualised under an inverse fluorescence microscope.

The aim of this study was to use µCP to label microglia in organotypic brain slices with the Iba1 antibody and visualise them via live-cell imaging. We show that microglia can be labelled with Iba1 and CD11b antibodies and followed with live-cell imaging for up to 20 days. Incubation in LPS or GM-CSF stimulated the migration of round microglia, while IL-10 promoted their differentiation into ramified forms.

## 2. Methods

### 2.1. Organotypic Brain Slices

**Figure 1:**
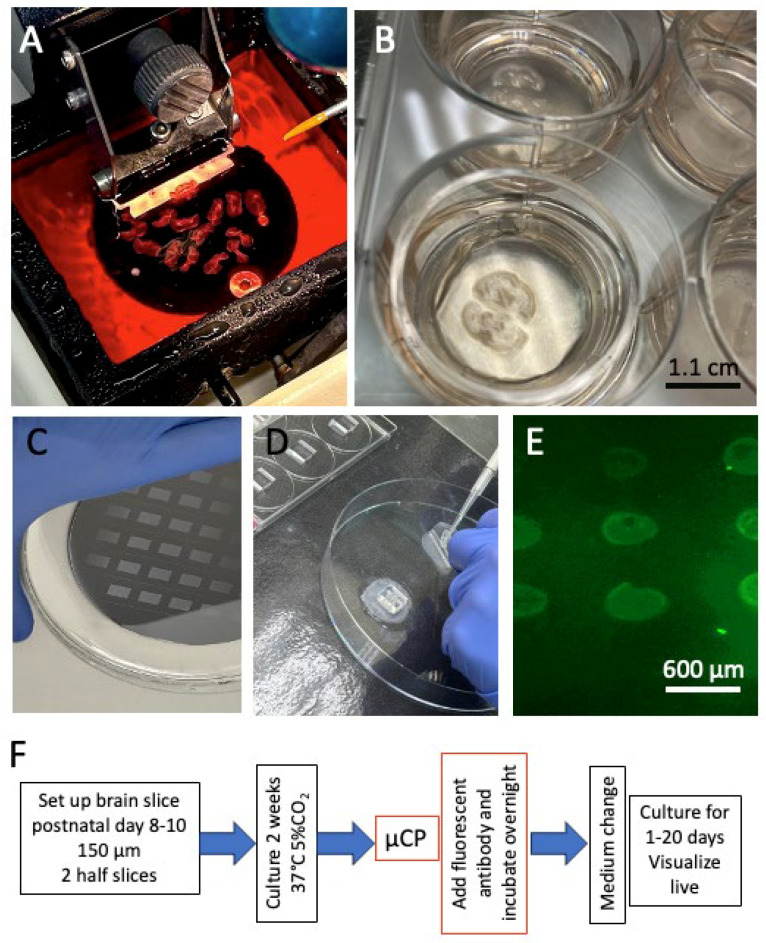
Culture of mouse organotypic brain slices. The brains of postnatal day 8–10 mice are sectioned (**A**) using a vibratome (150 µm), and two hippocampal half-brain slices are positioned on a semipermeable membrane in a membrane insert containing a 6-well plate and cultured for 2 weeks (**B**). Stamps are produced from a master (**C**) and placed on the brain slice (**D**). Figure (**E**) shows a positive control of a print (3 × 3 400 µm spots) of a fluorescent green Alexa-488 antibody on a membrane. Figure (**F**) shows a scheme of the experimental set-up. Scale bar = 1.1 cm (**B**) and 600 µm (**E**).

The culturing of organotypic brain slices has been well documented in our lab [20]. Brain slices (150 µm thick) are prepared from day 8–10 postnatal C57BL6 mice using a vibratome (Leica, Wetzlar, Germany, VT1000S) (Figure 1) in cooled conditions (~5 °C, Julabo F250 cooling system, Seelbach/Germany). In this study, hippocampal half-brain slices were obtained (Figure 1B) and placed on Biopore membranes (10FT roll, BGCM0010, Merck-Millipore) in 0.4 µm membrane inserts (Merck Millipore, PICM03050). The slices were cultured (Figure 1B) in 6-well plates (Sarstedt, 83.3920) with the slice medium comprising Minimal Essential Medium (16.1 g/L; MEM, Gibco, 11012044) supplemented with NaHCO_3_ (0.43 g/L), glucose (6.25 g/L; Merck, 8342), glutamine (116 mg/L; Merck, 1.00289.0100), 10% horse serum (Gibco, 16050-122, Lot: 2320064), 25% Hanks’ Balanced Salt Solution (HBSS, Gibco, 24020091), and a 1× antibiotic–antimycotic solution (Gibco, 15240-062) that was adjusted to a pH of 7.2. Slices were cultured in a CO_2_ incubator at 37 °C (5%) for up to 20 days, and the medium was replaced weekly. All experiments adhered to the principles of the 3Rs (replace, reduce, and refine), were approved by the Austrian Ministry of Science and Research, and complied with Austrian animal welfare guidelines.

### 2.2. Microcontact Printing of Iba1 onto Brain Slices

Live-cell labelling in brain slices has recently been described in detail [17]. Briefly, polydimethylsiloxane (PDMS) stamps were generated using silicon wafer templates (“master” moulds). Each master mould (3 × 3 with 400 µm diameter dots) was bought from GESIM (Ges. für Silizium-Mikrosysteme, Großerkmannsdorf, Germany), enabling the production of 38 stamps (Figure 1C). The stamps were sterilised under UV light for 10 min. A collagen mix was prepared by combining 66.7 µL of a type I bovine collagen solution (3 mg/mL, Sigma, 804592), 10 µL of 10x phosphate-buffered saline (PBS), 0.8 µL of 1 N NaOH, and 10 µL of the rabbit anti-Iba1 antibody (WAKO Code 019-19741, Fujifilm, Ratingen, Germany) in PBS. Then, 0.5 µL of this collagen solution was carefully applied onto four of the 400 µm dots and incubated at 37 °C in a sterile chamber. Next, a PEG stock solution (2.5 mg 4arm-PEG succinimidyl succinate in 200 µL PBS, Sigma-Aldrich, JKA7006) was prepared, and the Biopore membrane was placed in a Petri dish with the slices facing upward. After orienting the slice, 100 µL of the PEG solution was added beneath the Biopore membrane containing the slices. The collagen-loaded PDMS stamp was laid over the slices, ensuring that each half-brain slice was covered with four dots (upper left and right; lower left and right). Next, a 4 g weight was placed on top of the stamp, which was then incubated at 37 °C for 15 min in a sterile chamber (Figure 1D). The weight was carefully detached from the slices, and the membrane with the slices was transferred back to the inserts in the 6-well plate and incubated with an anti-rabbit Alexa-488 antibody (1:400) in 1 mL of its medium overnight. The next day, the slices were washed by replacing the medium, allowing them to be used for further treatments and microscopy (see Figure 1F for the experimental set-up). As a control, 3 × 3 spots of a fluorescent green Alexa-488 control antibody were printed on a semipermeable membrane (Figure 1E). As a negative control, 5 µL Iba1 antibody was preabsorbed with 5 µL recombinant Iba1 protein (200 µg/mL; AIF1 protein human (C-His) HY-P7488, MedChemExpress) overnight, followed by µCP. In comparison, microglia were labelled after µCP with a rabbit anti-CD11b antibody (abcam ab128797). As an additional control experiment, microglia were also labelled with both antibodies through co-µCP: the rabbit anti-Iba1 antibody and a rat anti-CD11b antibody (abcam ab8878-1013). Briefly, both antibodies were added to the collagen solution and printed onto the slices; then, the Iba1 was labelled with an anti-rabbit Alexa-488 antibody, and, after washing, the CD11b was labelled with an anti-rat Alexa 555 antibody.

### 2.3. Live-Cell Imaging

The slices (facing down) on the Biopore membrane were placed in 100 µL of sterile PBS and were investigated under an inverse fluorescence microscope. Images were captured at 2×, 4×, or 10× magnification using a Bresser MicroCam pro HDMI camera with an exposure time of 120 msec and a gain of 30. After imaging, the slices were transferred back to the original membrane insert for continued incubation, enabling long-term imaging for up to 20 days.

### 2.4. Stimulation of Slices

One day after microcontact printing and labelling (2 weeks in culture), the slices were stimulated with 0.1–1 µg/mL lipopolysaccharide (LPS), 10–100 ng/mL granulocyte–macrophage colony-stimulating factor (GM-CSF), or interleukin-10 (IL-10). Negative controls were treated with PBS under identical conditions.

### 2.5. Propidium Iodide (PI) and DAPI Staining

PI is a red fluorescent dye that specifically and easily penetrates dying cells and stains nuclei. To analyse cell viability, slices were incubated in 2 µg/mL PI while they were alive. The slices were then fixed with 4% paraformaldehyde for 60 min, washed, and counterstained with fluorescent blue nuclear DAPI for an additional 60 min. Subsequently, the slices were washed according to the staining protocol and analysed through fluorescence microscopy to assess cell death.

### 2.6. Data Analysis and Quantitative Analysis

Microscopic analysis was performed using a Leica DM IRB inverse fluorescence microscope with long-working-distance objectives. Green fluorescence (Alexa-488) was visualised using the L5 filter (excitation 480/40; cutoff 505; emission 527/30), while red fluorescence (Alexa-546) was visualised using the Y3 filter (excitation 535/50; cutoff 565; emission 610/75). Amoeboid cells were counted when cells were round with no processes, and differentiation (ramified cells) was defined as a morphological transformation involving at least 1 process, while macrophages had a large cell body of at least >50–70 µm. Statistical analysis was performed using One-Way ANOVA followed by a Fisher LSD post hoc test, where *p* ≤ 0.05 represents significance.

## 3. Results

**Figure 2:**
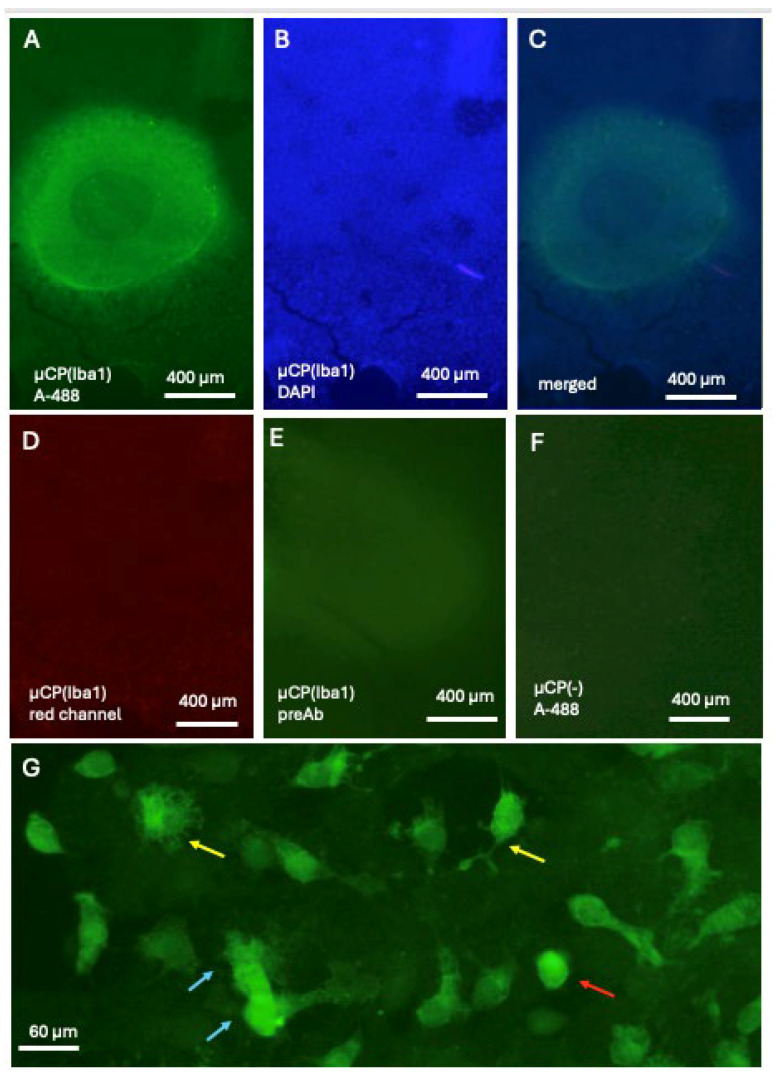
Microcontact printing (µCP) of Iba1 shows strong fluorescent green Alexa-488 (A-488) microglia in a round spot (**A**). The spot is localised over brain tissue stained with fluorescent blue nuclear DAPI (**B**). Note that DAPI+ nuclei are seen inside and outside the spot (merged picture (**C**)). No staining is seen in the same slice in the red channel, showing the specificity of green fluorescent Iba1 µCP (**D**). When the antibody was preabsorbed with recombinant Iba1 protein (preAb) and then µCP, only background staining is seen (**E**). As an additional control, slices underwent µCP without the Iba antibody (µCP(−)) and showed only background staining (**F**). Three types of Iba1+ microglia are seen, round amoeboid (red arrow in (**G**)) and low and high ramified forms (yellow arrows in (**G**)), and very rarely larger macrophage-like cells (blue arrows in (**G**)). Scale bar = 400 µm (**A**–**F**), 60 µm (**G**).

Iba1 was microcontact-printed directly onto a half-brain slice and showed a round, strongly immunoreactive spot with a central “spot of the stamp” (Figure 2A) located on healthy brain tissue, visualised using fluorescent blue DAPI+ nuclei (Figure 2B). This staining was consistent and was seen on the µCP spot but also in tissue around the spot (Figure 2C). No staining was seen in the red channel, displaying the positive staining of green fluorescent Iba1 (Figure 2D). In order to show specificity, the antibody was preabsorbed with recombinant Iba1 protein and then µCP, and these slices showed only background staining (Figure 2E). As an additional control, slices underwent µCP without Iba antibody, and these slices also displayed only background staining (Figure 2F). Two weeks after µCP of Iba1 antibody, three different Iba1+ microglial forms were found, round amoeboid and ramified forms and large macrophage-like cells (Figure 2G).

**Figure 3:**
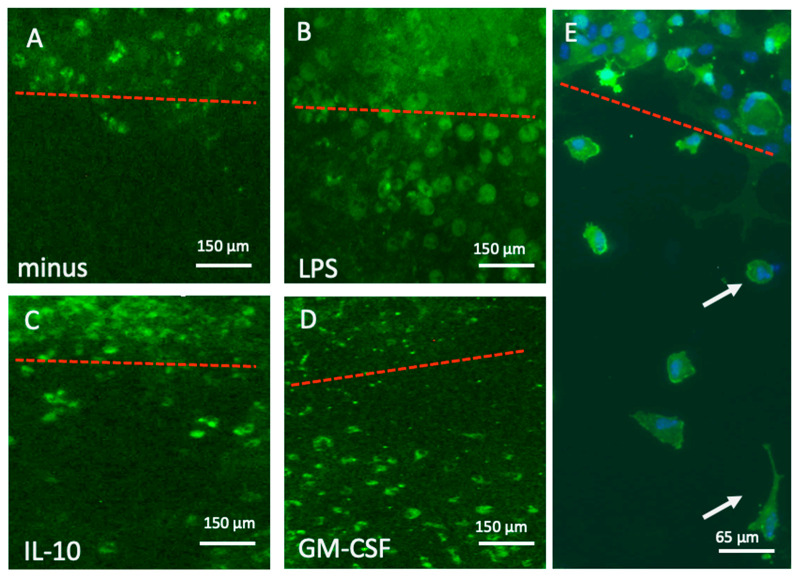
Migration of Iba1+ microglia into the space between brain slices after 10 days when incubated without (minus, (**A**)) or with 1 µg/mL lipopolysaccharide ((**B**), LPS), 10 ng/mL interleukin-10 ((**C**), IL-10), or 10 ng/mL granulocyte–macrophage colony-stimulating factor ((**D**), GM-CSF). Figure (**E**) shows a higher magnification of round (upper arrow) and ramified (lower arrow) microglia. The cells in Figure (**E**) were counterstained with fluorescent blue nuclear DAPI. The red dotted line shows the border of the slices. Scale bar = 150 µm (**A**–**D**) and 65 µm (**E**).

When slices were incubated without any exogenous substance in the medium, almost no Iba1+ microglia migrated out of the spots into the space (Figure 3A). Treatment with LPS markedly enhanced the migration of round amoeboid Iba1+ microglia (Figure 3B), while treatment with IL-10 (Figure 3C) and GM-CSF (Figure 3D) significantly enhanced the differentiation and migration of ramified Iba1+ microglia. The close-up view shows that Iba1+ microglia migrated in the space between the two brain slices (Figure 3E).

**Figure 4:**
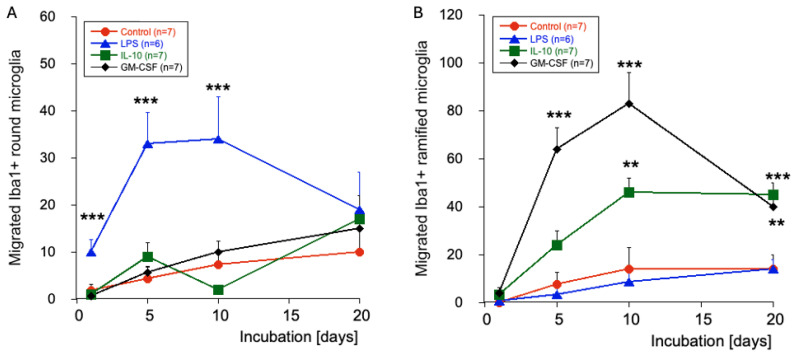
Quantitative analysis of the migration of Iba1+ microglia into the space between brain slices. Slices were labelled with the Iba1-Alexa488 antibody and then incubated and visualised after 1–5– 10 and 20 days. Slices were incubated without (red circle, control) or with 1 µg/mL lipopolysaccharide (blue triangle, LPS), 10 ng/mL interleukin-10 (green boxes, IL-10), or 10 ng/mL granulocyte–macrophage colony-stimulating factor (black triangles, GM-CSF). Values are the mean ± SEM of the total migrated cells per field (0.3 mm^2^); the n number is given in the box. The left panel (**A**) shows migrated round microglia, while the right panel (**B**) shows migrated ramified microglia. Statistical analysis was performed using One-Way ANOVA followed by a Fisher LSD post hoc test compared against the results for day 1. ** *p* < 0.01; *** *p* < 0.001.

The quantitative analysis of migrated Iba1+ microglia over 20 days shows almost no round amoeboid and ramified Iba1+ microglia in the space between brain slices (Figure 4). When the slices were incubated with LPS, the round amoeboid microglia started to migrate from day 1; this migration was most prominent after 5 and 10 days but decreased after 20 days (Figure 4). While IL-10 or GM-CSF treatment did not enhance the migration of round amoeboid microglia (Figure 4), when slices were treated with GM-CSF, a high number of ramified microglia were seen in the space between slices after 5 and 10 days (Figure 4). Incubation with IL-10 similarly increased the migration of ramified microglia, though less potently (Figure 4). Interestingly, although the number of ramified microglia further decreased after 20 days, it was still higher than at the beginning (Figure 4). Regarding the large macrophages in the space between slices, their numbers were very low (0.25 ± 0.2 per field, *n* = 8) after 20 days of incubation and did not change after treatment with LPS (1.4 ± 0.4, n = 7), IL-10 (0.25 ± 0.25, n = 4), or GM-CSF (0.5 ± 0.3, n = 4).

**Figure 5:**
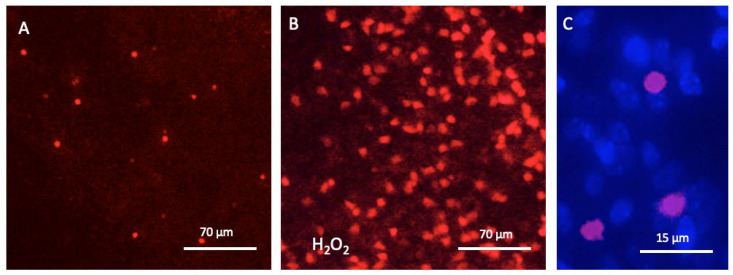
Cell death analysis with propidium iodide (PI). Two-week-old slices were microcontact-printed, incubated for 20 days, and then stained for viability with 2 µg/mL PI before being analysed. Panel (**A**) shows very few PI+ fluorescent red nuclei within the area of a (Iba1+) microcontact print. As a positive control, slices were incubated for 2 days with hydrogen peroxide, showing a dramatic increase in cell death (**B**). Panel (**C**) shows a high-magnification image of PI+ nuclei counterstained with fluorescent blue nuclear DAPI. Scale bar = 70 µm (**A**,**B**) and 15 µm (**C**).

In order to demonstrate the viability of cells in the µCP area, PI staining was performed. For the µCP spots of cultured slices (printed with Iba1), the level of PI staining was very low (2.25 ± 0.8 PI+ cells per 15,000 µm^2^, n = 8), indicating a very low degree of cell death (Figure 5A). As a positive control, slices were incubated with hydrogen peroxide (Figure 5B). Figure 5C shows an overlay of DAPI+ and PI+ nuclei and shows that not all cells were PI+ after treatment with hydrogen peroxide (Figure 5C).

**Figure 6:**
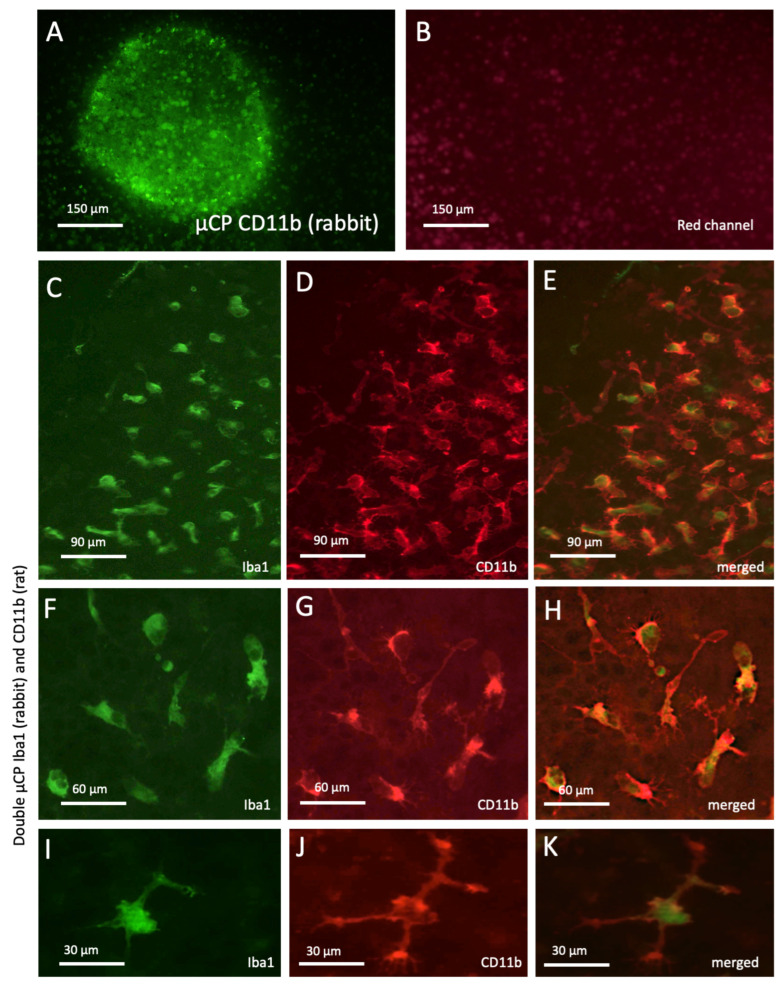
CD11b+ microglia: Microcontact printing (µCP) shows that Iba1 and CD11b were positively labelled in the brain slices. Figure (**A**) shows a 400 µm spot with fluorescent green CD11b+ microglia (made in rabbits) in brain slices after microcontact printing (**A**). No staining was seen in the red channel of the same field, showing specificity (**B**). In order to prove specificity, Iba 1 and a CD11b antibody (made in rats) underwent double µCP and were stained with fluorescent green Alexa-488 (Iba1, (**C**,**F**,**I**)) and fluorescent red Alexa-555 (CD11b, (**D**,**G**,**J**)). Figures (**E**,**H**,**K**) show the merged pictures, where Iba1 appears cytoplasmic, while CD11b is expressed extracellularly at the cell membranes. Scale bar = 150 µm (**A**,**B**), 90 µm (**C**–**E**), 60 µm (**F**–**H**), and 30 µm (**I**–**K**).

In order to prove specificity and act as an additional positive control, microglia underwent µCP with the microglial marker CD11b (made in rabbits). CD11b markedly labelled microglia, with labelling being more potent and pronounced in a round spot (Figure 6A). This staining was specific, as it was not seen in the red channel (Figure 6B). In order to further prove that Iba1 and CD11b were identically labelled, a double µCP was performed with both the Iba1 antibody and a CD11b antibody (made in rats). Indeed, Iba1 and CD11b co-localised in the majority of microglial cells (Figure 6C–K). We quantified three slices with a total of eight spots comprising 55 ± 7 cells and found that 80.5 ± 2.1% of the cells were co-stained with Iba-1 and CD11b.

**Figure 7:**
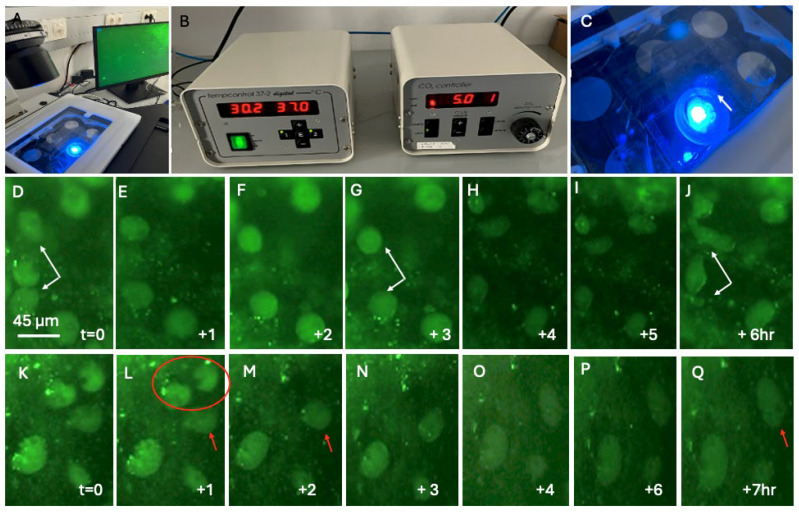
Live-cell imaging of Iba1+ microglia. Brain slices were cultured in a chamber connected to an inverse fluorescence microscope at 37 °C and at 5% CO_2_ (**A**–**C**). The membrane with the slices was placed in a 6-well plate with the slice facing down and visualised under fluorescence light (arrow in (**C**)). Panels (**D**–**J**) point to two Iba1+ microglia (white arrows in (**D**,**G**,**J**)), which show morphological changes after 6 h (**J**). One cell between these two microglia disappeared after 1 h (**E**–**G**). Panels (**K**–**Q**) point to two Iba1+ microglia (red circle in (**L**)) that disappeared within 2 h, while one cell (red arrow in (**L**,**M**,**Q**)) underwent a morphological transformation within 7 h (red arrow in (**Q**)). Note that differentiation (a morphology transformation with at least one process) was not observed over such a short time period (7 h). Scale bar = 45 µm (**D**–**Q**). Note that the scale bar in Panel D is the same for all other panels (**E**–**Q**).

To track microglia over a longer period, we used a culture system connected to a fluorescence microscope (Figure 7A–C). Brain slices were incubated in a chamber (Figure 7C) at 37 °C and at 5% CO_2_ (Figure 7B). Figure 7 shows two examples of Iba1+ microglia followed for 6–7 h after stimulation with GM-CSF. Figure 7D–J show three Iba1+ microglia, where one cell disappeared after 1 h, while the other two show some forms of morphological transformation after 6 h. Figure 7K–Q show two microglia that disappeared after 2 h and one cell with a clear morphological transformation after 7 h. Note that differentiation (a morphological transformation with at least one process) was not observed over such a short time period (7 h).

## 4. Discussion

In the present study, we were able to label microglia in organotypic brain slices with Iba1 and CD11b antibodies using direct microcontact printing. We followed these fluorescently labelled microglia for up to 20 days and showed migration as well as differentiation through live-cell imaging.

### 4.1. Organotypic Brain Slices and Viability

Organotypic brain slices represent a three-dimensional brain model that allows us to study several brain cell types ex vivo [18,29,30,31]. These slices are generally cultured on semipermeable 0.4 µm pore inserts between a humidified atmosphere and a culture medium, where the slices attach to the membrane [20]. Using brain slices has been a well-established practice in our research group for more than 20 years. We have studied neurons, astrocytes, microglia, and vessels from different brain areas, such as the hippocampus, striatum, basal nucleus, and mesencephalon [18]. Usually, slices flatten down and are transparent, which is a good sign of healthy slices. In the present study, we connected two half-brain slices from postnatal mice and positioned them 2 mm apart to study the migration and differentiation of microglia. The rationale for connecting these slices was to study migration and differentiation directly in the space between the two half-brain slices. A razor cut on the slice allows for better migration, as no glial slice scar inhibits cell migration. No effects were tested in other areas of the slice. Unfortunately, the culturing of older mice (>P14), adult mice, or human tissue for organotypic brain slices is limited due to the low viability of cells [19]. More work will be necessary to translate these findings to adult brain slices.

### 4.2. Microcontact Printing on Slices

We have used collagen-loaded µCP for several applications: to load growth factors or Alzheimer-related molecules of interest, to study the outgrowth of different brain cells along µCP lanes, to develop brain-on-a-chip technology [28], and to develop a live-cell imaging method [17]. In order to label brain cells, we press a specific antibody directly onto the brain slice using a master stamp. So far, we can label cells with GFAP, laminin, and now Iba1 and CD11b. Since these antibodies also label intracellular antigens, we believe that a collagen-mediated process together with some forms of “trauma” or “stress” cause antibody uptake, along with an increased membrane permeability and activation of the cells. This could happen in a similar way to the process described for the lipid-mediated uptake of proteins. However, so far, we have not yet discovered the exact mechanism of this process. In our previous original set-up [17], while we were able to optimise the weight-induced pressure (4 g), time (15 min), and temperature (37 °C) and found reproducible spot sizes and labelling densities, we did not analyse detailed quantitative metrics. While the stamp may damage parts of the brain slice, the procedure did not induce dramatic cell death, as shown through propidium iodide labelling, and the cells were viable even after several weeks of incubation [17]. The quality of this technique is mainly dependent on the quality of the brain slice, which is the greatest source of difficult in this method. The slice is a multi-layer cell model, and the age of the mice used is essential, as it influences the thickness, flattening, and quality of the slices. It also must be mentioned that this technique allows us to easily follow cells at the surface of the slices but not cells at deeper vertical layers.

### 4.3. Iba1 and CD11b Microglia in Slices

We and others have extensively studied microglia in organotypic brain slices [21,22,23,24,25,26]. Iba1 (ionised calcium-binding adaptor molecule 1) is a well-established marker for microglia that enables the detection and characterisation of their morphology, including processes with actin bundles and phagocytosis. We used this antibody in the present study and showed selective and specific labelling for all three kinds of microglia: round amoeboid, ramified, and large macrophages. We demonstrated that Iba1^+^ microglia cells survive in organotypic brain slices even after long-term incubation periods in vitro. After a 3-week incubation period, microglia displayed a healthy cytoplasm, processes, and nuclei, making them useful for further testing in our slice model. This is also consistent with other studies from our lab, which showed a preserved cell architecture in slices [17].

The results were further supplemented with a second microglial-specific antibody, CD11b, and we observed healthy, differentiated microglia, confirming that they were properly stained and remained healthy despite a relatively rough microcontact printing, staining, and culture period. In the present study, we started with Iba1, as this is a well-known microglia-specific antibody that has been well characterised in our lab for several years. We confirmed this microglial staining using a rabbit CD11b antibody. In order to show specificity and co-staining, a double µCP with the Iba1 antibody and a second CD11b (rat) antibody showed that the majority of labelled cells (80.5 ± 2.1%) were identical for both markers. The double µCP also showed the intracellular staining of Iba1, while more CD11b remained at the cell membranes.

### 4.4. Effects of LPS, GM-CSF, and IL-10

The endotoxin LPS is a well-established molecule for inducing chronic inflammation in vitro or in vivo [32,33] and activates the production of different pro-inflammatory cytokines from activated glial cells. The addition of LPS caused a dramatic microglial migration event, which is consistent with the results seen in other studies [34]. These activated amoeboid-shaped microglia can migrate to the injury site [35]. Furthermore, the microglia migrated in a “cluster-like” pattern that was probably caused by the inflammatory nature of LPS, similar to the aggregation of microglia around beta-amyloid plaques [36,37].

Granulocyte–macrophage colony-stimulating factor (GM-CSF) is found in many differentiated and non-differentiated cell types, including T cells, monocytes, macrophages, fibroblasts, and endothelial cells [38,39,40]. In this study, while an increase in round microglial migration was observed, accompanied by increased ramification, no large macrophages were observed. This finding is supported by the findings of other researchers [38,39,40], who showed an increased proliferation with fewer ramification events and that GM-CSF increased cell proliferation and microglial activation [38,39,40].

Interleukin-10 (IL-10) is an anti-inflammatory cytokine and is secreted by monocytes or TH2 lymphocytes [41,42,43]. It has several functions in the immune system and has strong anti-inflammatory effects, protecting the brain against extensive inflammation [41,42,43,44]. In the present study, the addition of IL-10 resulted in a greater number of migrated ramified microglia. This finding concurs with other studies, which reported that the absence of IL-10 caused an increased shift to round microglia and that IL-10 increased ramification and anti-inflammatory responses [41,42,43,44].

### 4.5. Migration Capacity

The migration of microglial cells in the brain environment is an essential physiological strategy by which microglia enter brain areas that are damaged [45,46,47]. Round amoeboid microglia have the capacity to quickly migrate into the brain, e.g., to reach areas that are damaged after traumatic injury or to phagocytose beta-amyloid plaques in AD-affected brains. This feature occurs due to the fact that microglia originate in the foetal yolk sac [48]. In the present study, we show that Iba1^+^ round amoeboid microglia can migrate quickly, especially when stimulated with LPS. This model offers a convenient method for investigating the migration capacity of living microglia. Cell migration is essential for the physiological function of the brain, as it coordinates cortical layer formation and is responsible for tissue homeostasis [49,50]. Migration is a complex system involving effectively timed and spaced chemical signals [51]. The mechanisms of migration involve membrane receptor activation, cytoskeleton remodelling, and environmental cues. The dysregulation of these migration mechanisms can lead to chronic inflammation and subsequent neurodegeneration [52].

### 4.6. Differentiation Capacity

Microglia have the capacity to differentiate very effectively, have a high morphological and functional plasticity, and can differentiate into ramified forms [53,54,55]. As microglia have two different, opposite functions, they are considered a “double-edged sword”: they can be protective or toxic [56]. If microglia detect damaged tissue, they can produce and secrete trophic growth factors or anti-inflammatory factors to induce neuronal repair. If sensory cells in the brain detect cell death or plaque deposition, they produce pro-inflammatory cytokines and differentiate into macrophages to eliminate debris. This is an important feature in the AD-affected brain for eliminating large beta-amyloid plaques. However, microglia lose their phagocytic capacity and become dysfunctional in the brains of patients with severe AD [57]. Our model offers a convenient method for studying not only migration capacity but also subsequent phagocytosis in a living environment. In this study, differentiation was defined as a morphology transformation involving at least one process. The different phenotypes of differentiation also depend on their environment and external signals , as LPS almost completely shifted all microglia into their round ameboid form, while GM-CSF induced microglial ramification. However, in this study, differentiation could not be observed through live-cell imaging within the short time period of 7 h.

### 4.7. Live-Cell Images: A Proof-of-Principle Model

In a recent study [17], we presented an innovative method to label and visualise astrocytes and vessels in living slices. Using microcontact printing, astrocytes were labelled with GFAP, while vessels were labelled with laminin. This approach is cost-effective and easy to use with a conventional inverse long-distance fluorescence microscope. In the present study, we extend our findings and show for the first time that Iba1+ (and CD11b+) microglia can be visualised using live-cell imaging, which is a potent tool for studying the migration and differentiation of microglia. We demonstrated proof of this principle, as it was possible to reliably locate and observe the same cell for up to 7 h. This is consistent with other studies, confirming that a collagen environment is suitable for elucidating cellular dynamics in live-cell imaging [58]. Furthermore, live-cell imaging following a similar methodology has already been used to study collagen in living cells [59]. Right now, we aim to improve the system by using a pump to continuously deliver medium to the slices, by observing the same cells over a period of 7–14 days, and by compiling a short movie. This is very difficult, as the pump is connected to a culture chamber directly in the microscope and must be triggered so that the slices (cultured now on a novel ring insert) do not dry out and so that not too much of the medium, which can easily evaporate, is added. Furthermore, a better camera system with live video tracking may increase the number of live-tracked cells and provide more accurate data. Additionally, there are other well-established and potent methods for visualising live microglia: for example, brain slices can be prepared from transgenic mice possessing a microglia-specific fluorescent reporter gene. In such a model, genes can be turned on or off and visualised. The only disadvantage is that, for such experiments, transgenic mice must be bought and bred, which is a strict and costly process, and slices cannot be prepared from adult mice. Alternatively, fluorescent reporter genes could be transferred directly into the brain slice, but this process is very complex and only works with viruses or gene guns. The use of viruses involves strict working under safety environments, and gene guns are very expensive, especially when using gold particles. At present, using nanoparticles or pan-surface antibodies may be an alternative method, though selective penetration into the target is difficult and requires extensive optimisation.

### 4.8. Limits of This Study

Although this novel live-cell imaging technique offers the first proof-of-principle method for detecting microglia over 20 days, it is not without its limitations. First, the printing field of 400 µm is large, and commercial automated laser-assisted systems may improve the model. Second, while we were able to label and follow vessels, astroglia, and now microglia, triplicate labelling remains untested. The interaction between these cell types over weeks will be a new aim to follow up on. Third, long-term cell survival (over weeks) was also not systematically evaluated. In the future, we will couple brain slices to a microscopic chamber and a pump to deliver the medium so that we can follow brain cells without transferring them back into the incubator. Fourth, applying stamps directly onto brain slices can exert mechanical stress and potentially damage the slices. To mitigate this, the printing technique must be optimised. Fifth, even though the team at our lab is very skilled at making organotypic brain slices, several slices were discarded because they did not look healthy . This occurred because the microcontact printing process is rather rough on the slices and partly damages them, especially in regions close to the space between slices. Ensuring the correct orientation of the spots directly along a slice’s edges is also not trivial. Sixth, this study only differentiated cells by phenotype, and we did not explore any functional and morphological state of the microglia in the printed compared to the non-printed (untouched) areas. In future studies, the effects of this long-term binding of antibodies to endogenous proteins should also be tested, along with any potential undesired side effects. Finally, the microglia were counted by eye under a microscope, and quantification using machine learning techniques would lead to quicker and more reliable results.

## 5. Conclusions

Overall, the present study shows for the first time that microglia can be labelled in mouse organotypic brain slices using microcontact printing with microglia-specific antibodies. These fluorescently labelled microglia can be visualised for up to 20 days through live-cell imaging. This method is straightforward, allowing us to effectively label all cell types in the brain, such as astroglia, vessels, and now microglia. We show that microglia migrate out of the brain slice and can differentiate to some extent. Microglia are very flexible in shape and function, and this new method may allow us to investigate novel aspects of microglial activity.

## Data Availability

Data are available upon request.
